# Clinical Experience of Therapeutic Plasma Exchange (TPE) in Severe Leptospirosis: A Case Series from Sri Lanka

**DOI:** 10.3390/tropicalmed11050132

**Published:** 2026-05-12

**Authors:** Manana Dewage Sankani Vishvara Kularathna, Senanayake Abeysinghe Mudiyanselage Kularatne

**Affiliations:** 1 Teaching Hospital Peradeniya, Peradeniya 20400, Sri Lanka; 2 Department of Medicine, Faculty of Medicine, University of Peradeniya, Peradeniya 20400, Sri Lanka; tbn1917@gmail.com

**Keywords:** leptospirosis, pulmonary haemorrhages, myocarditis, therapeutic plasma exchange

## Abstract

Background: Leptospirosis is a globally prevalent zoonosis with significant morbidity and mortality, especially in tropical regions like South Asia. In its severe form, the disease often leads to multiorgan dysfunction, with pulmonary haemorrhage being a major cause of death. Evidence supporting specific treatments for severe leptospirosis with pulmonary involvement remains limited. Recent studies suggest that immunomodulatory therapies, such as therapeutic plasma exchange (TPE), may offer survival benefits. This case series explores the application and outcomes of TPE in patients with severe leptospirosis at a tertiary care hospital in Sri Lanka. Methods: We studied a case series involving nine patients with confirmed severe leptospirosis and multiorgan involvement from September 2021 to October 2022. All patients received standard care, including intravenous antibiotics and methylprednisolone. TPE was initiated in all nine patients based on clinical severity, particularly in the presence of pulmonary haemorrhage. Clinical, laboratory, and radiological data were collected from patient records and follow-up. Leptospirosis diagnosis was confirmed through ELISA IgM testing. TPE decisions were made by a multidisciplinary team. Results: Of the nine patients who received TPE, seven survived (78%). Pulmonary haemorrhage was the primary indication for TPE in all cases. All patients had multiorgan involvement: renal failure (89%), hepatic dysfunction (55%), and myocarditis (67%). Mortality was associated with inotropic-dependent myocarditis and mechanical ventilation at TPE initiation. Patients requiring intubation had a 50% mortality rate, compared to 14% in those who were not intubated. Non-survivors also had significantly elevated lactate levels (>4 mmol/L) and worsening acid–base status. Four patients required dialysis: three survived. Conclusions: Early initiation of TPE may be safe and beneficial in severe leptospirosis, and future randomised controlled studies are necessary to examine its benefits further. These findings are hypothesis-generating for further research, particularly on patient selection for TPE.

## 1. Introduction

Leptospirosis has been recognised as the most widespread zoonotic disease globally [1]. It infects 1 million people and causes 58,000 fatalities and 2.9 million disability-adjusted life years annually worldwide [2]. The disease is particularly prevalent in South Asia, the Asia Pacific, and Latin America [3].

Sri Lanka has experienced several significant outbreaks over the past few decades, including a major outbreak in 2008, during which 7421 cases were reported to the national Epidemiology Unit [4]. The case fatality rate during that outbreak was 3%.

Transmission of the infection to humans occurs through direct contact with infected animal tissue or bodily fluids, or indirectly via environmental contamination, with the pathogen entering through breaches in the skin or mucosal surfaces [1,5].

The clinical manifestations of leptospirosis in humans can vary from a mild, self-limiting acute febrile disease to a severe, life-threatening disorder involving multiple organ dysfunction [6]. Acute kidney failure, pulmonary haemorrhages, myocarditis, pancreatitis, and multiorgan dysfunction syndrome are among the severe, life-threatening systemic manifestations [4]. A proportion of infected individuals will develop severe illness with multi-organ failure for reasons that remain inadequately explained. Current understanding suggests that both pathogen-related factors (infecting serovar/species, inoculum size) and host-related factors (immunological response) contribute to this variation [7].

Pulmonary involvement in leptospirosis has a high case fatality rate and is the predominant cause of mortality associated with the disease. Immune pathways are pivotal in the pathophysiology of leptospiral pulmonary haemorrhage [8]. Evidence for effective treatment methods for severe leptospirosis with pulmonary haemorrhages is limited [8]. Immunomodulation with plasma exchange, intravenous immunoglobulin, and/or glucocorticosteroids has been used in severe leptospirosis [9]. There are reports that indicate the safe application of plasma exchange in leptospirosis [5,10]. Our report describes a series of patients with severe leptospirosis who were admitted to the Teaching Hospital in Peradeniya (THP), Sri Lanka, over a 1-year period and treated with therapeutic plasma exchange (TPE), with beneficial outcomes observed in the majority of patients.

## 2. Methodology

### 2.1. Patients, Diagnoses, and Basic Treatments

The study comprised 9 patients with life-threatening severe leptospirosis and multi-organ involvement who were treated according to the guidelines for severe leptospirosis based on clinical experiences and practices of the hospital [11]. To assess the severity of the infection, the Sequential Organ Failure Assessment (SOFA) score was calculated [12]. All 9 patients received methyl prednisolone and plasma exchange as therapeutic modalities in addition to all supportive treatments provided in the Intensive Care Unit (ICU). The data were collected over a period of one year, both prospectively and retrospectively, from September 2021 to October 2022.

Information regarding social and demographic factors, clinical manifestations, examination results, investigations, and treatment was obtained through direct interviews with patients and their relatives during hospital stays and follow-up clinic visits, as well as from hospital records. The ELISA IgM test for leptospirosis was used to confirm the diagnosis in all 9 patients. The ELISA IgM kit was developed in-house by the Departments- Microbiology and Parasitology, Faculty of Medicine, University of Peradeniya, in collaboration with Japan.

Sociodemographics included age, gender, and area of residence, and clinical data included exposure history, premorbid health conditions, duration of fever, and additional systemic complaints such as dyspnoea, headache, vomiting, abdominal pain, bleeding, urine output, and examination findings such as pulse rate, blood pressure, and crepitations in the lung bases. Laboratory investigations included a complete blood count, liver and kidney biochemical profiles, serum electrolytes, troponin levels, venous blood gas analysis, chest radiography, and 2D echocardiography as warranted by the clinical presentation. These investigations were reviewed just before the TPE decision.

All patients were treated with intravenous antibiotics effective for leptospirosis, including ceftriaxone, doxycycline, and benzyl penicillin. All patients received IV methylprednisolone upon achieving a clinical severity score of 2, as described in the previous publication [11]. The treating physicians determined the choice to provide these therapies based on the clinical severity of the condition, including pulmonary haemorrhages and involvement of other systems. We included all patients with leptospirosis who underwent TPE during the study period.

### 2.2. Identification of Multiple System Involvement

The identification of pulmonary haemorrhages was based on respiratory distress, blood-gas-based type I respiratory failure, a drop in haemoglobin, or CXR findings of diffuse alveolar shadows. Involvement of other systems was based on appropriate investigations, including ECG, cardiac enzymes, serum creatinine, liver transaminases, and related parameters.

### 2.3. Management Protocol

Some patients received blood and platelet transfusions. Management decisions, including the application of TPE, were made on a case-by-case basis by a multidisciplinary team in the Intensive Care Unit (ICU). Other management included oxygen, intravenous fluids, antibiotics, and the detection and intervention of complications.

### 2.4. Therapeutic Plasma Exchange (TPE)

This procedure was performed only on an ICU bed, in accordance with the guidelines of the American Society of Apheresis (ASFA). The TPE machines (Fresenius Kabi AG, Homburg, Germany) utilised continuous flow, which follows automated centrifuge-based technology, and each procedure lasted approximately 3 h. The patient’s plasma was separated using an automated platform and replaced with 5% Albumin, Saline, or fresh-frozen plasma. The TPE procedure was performed by a consultant transfusion physician affiliated with the National Blood Transfusion Service (NBTS). For each patient, 2 or 3 TPE sessions were performed as required.

### 2.5. Data Analysis

The collected data were checked for any inconsistencies and errors, which were thereafter analysed using the 26th edition of the software, IBM SPSS Statistics.

## 3. Results

There were eight males and only one female patient (Table 1). The youngest patient was 25 years old, while the eldest was 65 years. All patients were from the Kandy District of the hilly Central Province. Eight patients had a significant history of exposure consistent with leptospirosis risk.

Patients were labelled chronologically for identification (Table 2). Four patients had no comorbidities, while two had diabetes (Patients No. 3 and 9), and one of these patients with diabetes had chronic kidney disease and ischaemic heart disease (Patient No. 3). Patient number 7 had bronchial asthma. None had chronic lung diseases, and information on cigarette smoking was not available.

Time from the onset of illness to hospital admission ranged from 2 to 10 days, with the majority (*n* = 5) presenting on the 3rd to 4th day. Patients were evaluated on admission according to the clinical severity score, which had been the practice of the managing team for leptospirosis [11]. Subsequently, the SOFA score was calculated to assess infection severity and organ involvement (Table 3). The median day of commencing TPE was day 5 of illness (range: 3–11 days).

### 3.1. Haematological Manifestations and Treatments

Eight patients (88.9%) exhibited haematological involvement, characterised by thrombocytopenia or a tendency for bleeding, including both non-survivors. Although frequently observed, haematological manifestations appeared reversible with early intervention, including platelet transfusion to prevent bleeding into vital sites such as the brain and lungs. Patients labelled 3, 4, 6, and 9 received both blood and platelet transfusions. Patients 2 and 5 received only blood transfusions, while patient 8 received only platelet transfusions. Of the two patients who did not survive, patient 9 received both blood and platelets, while patient 8 received only platelets.

### 3.2. Renal, Hepatic, Cardiac, and Pulmonary Manifestations

Renal impairment was evident in eight patients (88%) and hepatic dysfunction in five patients (55%). Renal and hepatic dysfunction together contributed to severe leptospiral disease and mortality. Two patients who died had both renal and hepatic dysfunction. Six patients (66%) had myocarditis, including two deceased patients. Only three (33%) patients had developed pulmonary haemorrhages on admission.

### 3.3. Pulmonary Involvement in TPE Decision

After admission, six more patients developed pulmonary haemorrhage, contributing to a total of nine patients at the onset of TPE, which was deemed the primary reason for the initiation of TPE. Table 3 shows the SOFA at the commencement of TPE. The SOFA score showed a rising trend from a median value of 11 on admission to 12 at TPE, and those who died had higher SOFA scores. The average duration from admission to TPE was 2 days (range: 1–4 days), indicating prompt commencement after clinical deterioration. TPE was generally conducted on day 5 of illness (median), coinciding with the onset of significant signs such as pulmonary haemorrhage. Among the nine patients who underwent TPE, seven survived, yielding a 78% survival rate.

### 3.4. Description of Fatal Cases

In both fatal cases (Patients 8 and 9), TPE was initiated on days 3 and 5, respectively, relatively early in the disease course. The fatality rate is 22% of the case series. Nevertheless, both patients exhibited significant multisystem involvement, including myocarditis, at the time of TPE, potentially limiting its therapeutic efficacy. These two patients deteriorated due to the progression of myocarditis-related complications. Both fatalities presented early in the illness (Day 2) and exhibited high severity scores at both admission and the initiation of TPE (Table 3). Patient 9, aged 65 years, was the oldest in the cohort, suggesting greater physiological vulnerability. Despite timely TPE on Days 3 and 5, both cases progressed rapidly, suggesting that in some patients, disease progression, myocarditis, and host factors, such as age and comorbidities, might reduce the benefit of TPE.

### 3.5. Haematological and Biochemical Parameters

Table 4 shows laboratory parameters on admission (A) and at the initiation of TPE (X). Six (60%) patients had haemoglobin (Hb) less than 10 g/dL, and minor variation of Hb% was observed between admission to the commencement of TPE in all patients. As expected, marked thrombocytopenia was found in all patients except cases 2 and 5. However, there was a noticeable further drop in platelet count at the beginning of TPE. Leucopenia was not a feature of all patients on admission, and high white cell count persisted at the commencement of TPE, except in case 8, where WBC dropped from 13 to 3.9 × 10^9^/L.

Severe acute kidney injury was prevalent. Importantly, creatinine levels over 500 µmol/L at the start of TPE did not predict death (for example, Patients 3 and 4 survived), suggesting that kidney injury alone may not lead to poor outcomes if treated promptly and effectively. Patients labelled 2, 3, 4, 5, and 9 showed significantly elevated AST and ALT levels (>200) at admission. Among them, only patient 9 died.

### 3.6. Arterial Blood Gas Variation

Arterial blood gas (ABG) results obtained at admission and before TPE were crucial in decision-making (Table 5 and Table 6). On admission, most patients (*n* = 8) demonstrated metabolic acidosis characterised by decreased bicarbonate levels and increased lactate concentrations. Eight patients were severely hypoxic. Survivors typically demonstrated stable or improved acid–base status and lactate clearance at the time of TPE. In contrast, both non-survivors (8 and 9) exhibited progressive acidemia, with pH decreasing from 7.35 to 7.13 and from 7.2 to 7.1, respectively, with an increase in lactate levels (up to 8.6 mmol/L). Five patients (1, 5, 6, 9, and 10) required intubation and ventilation with an FiO2 of 100%. Others also received supplementary oxygen.

### 3.7. SOFA Score and Outcome Predictors (Table 7 and Table 8)

The two patients who later died had the highest SOFA scores at presentation (both >14). Of the nine patients who underwent plasma exchange, three had myocarditis with inotrope dependency at the initiation of TPE. In the subgroup with myocarditis and inotrope dependency, two out of three patients (66.7%) died, both of whom were receiving three inotropic drugs at the onset of TPE. The only survivor in this group received two inotropes. In contrast, all six patients in the group without inotrope dependency at the initiation of TPE survived; three had myocarditis. The requirement for invasive mechanical ventilation at the commencement of TPE was associated with significantly increased death. In the group of five intubated patients, two died, resulting in a mortality rate of 40% within this category. Conversely, all four non-intubated patients survived, although three necessitated non-invasive ventilatory support (NIV/CPAP). Severe renal involvement required dialysis support for four patients, and of them, one died and three survived.

**Table 7:** SOFA Score on admission and number of deaths.

Clinical Score on Admission	SOFA Score on Admission	Number of Patients	Number of Deaths
3	<10	4	0
4	11–14	3	0
5	≥15	2	2

**Table 8:** Outcomes in patients with severe leptospirosis vs. supportive interventions.

	Deceased (2)	Survived (7)
Myocarditis with inotrope dependency at the beginning of TPE	2 (dependent on three inotropes)	1 (dependent on two inotropes)
No inotrope dependency	0	6
Intubated at the beginning of TPE	2	3
Not intubated at the beginning of TPE	0	4 (NIV/CPAP for three patients)
Needed dialysis during the course of the illness	1	3
Did not need dialysis during the course of the illness	1	4

NIV/CPAP = Non-invasive ventilation, continuous positive airway pressure.

## 4. Discussion

In this case series, the duration of the illness was short, and patients were ill with severe metabolic derangement and the therapeutic decision for TPE was taken within a few days after hospital admission. On admission to the hospital, patients had hypoxaemia and metabolic acidosis, which further deteriorated despite resuscitation. These metabolic derangements were due to multiple organ dysfunctions caused by leptospirosis. Reversing such severe manifestations is a greater challenge, particularly in an ICU setting without highly specific interventions. We found TPE to be a safe procedure for severe leptospirosis, but it should be used judiciously. Two patients died in this case series despite the application of all available treatment modalities, including TPE. Their common manifestations were the development of marked lactic acidosis, hypoxaemia, and myocarditis, which required ionotropic support to maintain blood pressure. Myocarditis may have developed early in these patients, causing them to feel unwell and to seek hospital admission on the second day of illness. In a state of cardiac instability, whether TPE may worsen it remains an open question and needs further evaluation. However, the anti-inflammatory action of steroids may help to control myocarditis in severe leptospirosis. The clinical decision regarding the use of these treatment modalities requires more evidence, including the timing of their use, as delays will affect outcomes.

Therapeutic plasma exchange has many applications in clinical practice [13]. TPE has two distinct mechanisms of action. Clearance of a pathogenic substance from the plasma and administration of substantial quantities of deficient plasma components [13]. Leptospirosis comprises two clinical phases: the septicemic phase and the immune phase. In this latter phase, the body’s humoral response functions to eradicate the organism from numerous tissues. Nonetheless, the deposition of immunological complexes during this period may result in endothelial injury [14]. A previous study indicates that membranous deposits of linear immunoglobulins (IgA, IgG, IgM) and complement on an alveolar surface may precipitate catastrophic pulmonary haemorrhage in leptospirosis in humans [15]. It is reasonable to suppress dysregulated immune responses before the initiation of immunologically mediated tissue destruction.

As leptospirosis is becoming a significant health problem with high mortality, clinicians in southern Sri Lanka use TPE for complicated leptospirosis. They found a beneficial outcome [10]. The limiting factor for TPE is the unavailability of the facility in rural farming regions where the disease is common. Our study highlights the value of TPE in the central region of Sri Lanka. This case series, while demonstrating the benefits of TPE, also highlights instances in which TPE has failed to provide survival benefits. The cumulative survival rate in this cohort was 78%; however, deceased patients had significant cardiac and pulmonary dysfunction. Although the timing of TPE fell within the early treatment window, the degree of systemic involvement may have reduced any possible positive effect.

Multiple system involvement is the nature of severe leptospirosis. The factors contributing to disease severity remain unclear, and the natural course of the illness is unpredictable. Therefore, clinical vigilance is essential. Three patients presented with pulmonary haemorrhages at admission, and subsequently, six additional patients experienced pulmonary haemorrhages that required TPE. Six patients had myocarditis on admission, and three patients necessitated inotropes to sustain blood pressure later in the course of the illness. Both patients who died required intubation due to severe respiratory distress with pulmonary haemorrhages and were diagnosed with myocarditis that needed the use of maximal inotropic support. This pattern suggests that lung haemorrhage and myocarditis are significant factors influencing negative outcomes in leptospirosis, particularly when occurring concurrently. The published literature also suggests that leptospirosis complicated by both pulmonary haemorrhage and myocarditis has a substantially poor prognosis, particularly in resource-limited settings or when multiple organ systems are implicated [16]. The elevated transaminases seen in several patients in the series are typical of severe leptospirosis: five patients had evidence of significant hepatic involvement, but there were no cases of fulminant hepatic failure. Elevated aspartate transaminase levels serve as a valuable prognostic indicator in advanced leptospirosis [17].

Elevated liver enzymes, particularly in conjunction with renal impairment or thrombocytopenia, may indicate impending organ failure and risk of bleeding. Furthermore, in contrast, the prevention of transaminase rise appears to be associated with improved survival following TPE. Marked thrombocytopenia was a common occurrence in these patients at admission, and platelet transfusions were needed in severe cases to prevent bleeding.

Hypoxaemia is a cardinal feature in these patients. There was a simultaneous deterioration in acid–base status, indicating ineffective oxygen utilisation. Patient 6, who presented with severe acidemia (pH 6.99), demonstrated notable improvement following supportive care, underscoring the potential for reversibility through early intervention. We think the presence of worsening acid–base imbalance and hyperlactatemia before TPE was linked to unfavourable outcomes. The findings indicate that ABG trends, specifically lactate and bicarbonate dynamics, serve as prognostic markers. The average initial blood lactate concentration in patients who died was markedly elevated compared to that of survivors (6.55 vs. 1.8 mmol/L). All survivors had lactate levels ≤2.6 mmol/L, whereas both non-survivors had levels exceeding 4 mmol/L—values typically associated with compromised perfusion and metabolic distress. In these cases, increased lactate levels likely indicate persistent circulatory shock or significant cellular hypoxia. Furthermore, a Brazilian multicentre retrospective study of 206 leptospirosis patients compared ICU-admitted and non-ICU-admitted patients, revealing that those admitted to the ICU exhibited metabolic acidosis (60.5% vs. 36.5%) and high mortality rates (23.5% compared to 5.7%) [18].

Patients with a high SOFA value showed a markedly higher mortality rate. These results support the contention that the timely initiation of TPE is safe and may have a beneficial effect before the development of respiratory failure requiring intubation.

We observed that the ventilatory state at the start of TPE had a significant impact on the outcomes. There were no deaths among patients who were not intubated, including those using non-invasive ventilation (NIV) or CPAP. Still, all documented deaths occurred among patients who were intubated before the commencement of TPE, with a mortality rate of 40% in this group. This pattern suggests that the need for invasive mechanical ventilation at the beginning of TPE may reflect greater disease severity and be associated with worse outcomes. However, the limited sample size precludes statistical significance. These results support the contention that the timely initiation of TPE is safe and may confer benefits before the development of respiratory failure requiring intubation. In a study conducted in Southern Sri Lanka involving 88 patients with complicated leptospirosis, logistic regression analysis identified intubation and mechanical ventilation as strong independent predictors of mortality, with an odds ratio of approximately 18.5 (*p* < 0.001) [16].

This study has limitations, as it is a case series, and the absence of a control group precludes rigorous statistical analysis and solid conclusions. This highlights the need for a control group in future studies. We used an IgM ELISA to confirm the clinical diagnosis, which has lower specificity than MAT and PCR, both of which were unavailable at the regional reference laboratory during the study period.

## 5. Conclusions

This study describes nine cases of severe leptospirosis at a single centre in Sri Lanka over one year and the utilisation of TPE judiciously to mitigate multiple organ dysfunction. The main impact of leptospirosis is felt in rural farming communities in Sri Lanka, where TPE facilities are unavailable. In this context, severe cases need to be transferred to TPE-capable centres in cities. To facilitate such an early referral system, educating medical professionals to detect severe cases using validated clinical criteria is a timely requirement. The findings of this case series will be useful for hypothesis generation and for conducting further research.

## Figures and Tables

**Table 1:** Demographic Characteristics of 9 patients.

Data	Distribution	Number, and %
Age	0–25 yrs	1 (11%)
	26–50 yrs	5 (55%)
	51–75 yrs	3 (33%)
Gender	Male	8 (88%)
	Female	1 (11%)
Significant exposure history (In muddy paddy fields)	Yes	8 (88%)
	No	1 (11%)

**Table 2:** General condition, multiple system involvement, system-based clinical severity (SOFA) at admission, and the day of application of TPE.

Patient No, Age, Gender.	1–53 M	2–49 F	3–57 M	4–37 M	5–40 M	6–44 M	7–43 M	8–25 M	9–65 M
Day of illness on admission	4	3	5	3	10	3	4	2	2
Day of illness at TPE	6	4	6	4	11	4	8	3	5
Poor general condition	+	+	−	−	−	+	−	+	+
Haematological	+	−	+	+	+	+	+	+	+
Pulmonary (SPHS)	−	−	+	−	−	+	−	+	−
Cardiac (Myocarditis)	+	+	−	−	−	+	+	+	+
Renal (Acute kidney injury)	+	−	+	+	+	+	+	+	+
Hepatic dysfunction	−	+	+	+	+	−	−	−	+
SOFA Score on Admission	10	9	11	11	9	13	7	15	19
SOFA Score at TPE	11	9	12	13	11	13	7	14	18
Co-morbidities	Epilepsy	HT	Diabetes, IHD, CKD				BA	Diabetes	
Outcome								Died	Died

Legend: + = Present; − = Absent; N/A = Not Applicable; TPE = Therapeutic Plasma Exchange, HT = Hypertension, IHD = Ischaemic heart disease, CKD = Chronic kidney disease, BA = Bronchial asthma, AD = Alcohol dependency, and SPHS = Severe pulmonary haemorrhagic syndrome.

**Table 3:** Sequential Organ Failure Assessment (SOFA) score on admission (A) and at the beginning of TPE(X).

Patient Number	Day of Illness on Admission (A)	Day of Illness at TPE (X)	SOFA Score (A)	SOFA Score (X)
1–53 M	4	6	10	11
2–49 F	3	4	9	9
3–57 M	5	6	11	12
4–37 M	3	4	11	13
5–40 M	10	11	9	11
6–44 M	3	4	13	13
7–43 M	4	8	7	7
8–25 M	2	3	15	14
9–65 M	2	5	19	18

**Table 4:** Comparative Laboratory Parameters on Admission (A) and at Initiation of Therapeutic Plasma Exchange (X).

Lab Parameter	Patient 1	Patient 2	Patient 3	Patient 4	Patient 5	Patient 6	Patient 7	Patient 8	Patient 9
Hb% (A)	9.5	9	8.9	12.3	9.9	11.6	12.3	9	11
Hb% (X)	9.2	9	9.5	10.1	7.5	10.8	11.4	11.1	9.7
Platelet count × 10^9^ (A)	39	123	20	30	104	41	87	47	30
Platelet count × 10^9^ (X)	34	123	14	33	89	24	50	59	24
WBC × 10^9^ (A)	10.9	9.4	9.5	10.4	14.9	3.8	18.47	13	14.08
WBC × 10^9^ (X)	13.1	9.4	16.3	11	17	3.8	11.9	3.9	10.7
Creatinine µmol/L (A)	213	121	532	606	270	239	236	108	303
Creatinine µmol/L (X)	200	121	532	606	270	239	101	149	201
K^+^ mmol/L (A)	5.1	3.1	5.9	4.3	5	4.5	3.68	3.4	4.7
K^+^ mmol/L (X)	4.9	3.1	6.1	5	4	4.5	3.8	3.49	5.9
AST U/L (A)	39	270	292	323	285	24	28.3	21.6	425
AST U/L (X)	36	282	292	323	296	24	39	26	400
ALT U/L (A)	24	132	152	119	158	28.5	55	24	371
ALT U/L (X)	22	145	152	119	148	28.5	84	34	342

Normal values: Hb (13–17 g/DL), WBC (4–11 × 10^9^), Platelet count (150–400 × 10^9^), Creatinine 62–166 µmol/L), K^+^ (3.5–5.1 mmol/L), ALT, and AST (0–40 U/L).

**Table 5:** Arterial Blood Gas Parameters on Admission (A) and at Initiation of Therapeutic Plasma Exchange (X).

Blood Gas Parameter	Patient 1	Patient 2	Patient 3	Patient 4	Patient 5	Patient 6	Patient 7	Patient 8	Patient 9
pH (A)	7.34	7.45	7.31	7.33	7.2	6.99	7.34	7.35	7.2
pH (X)	7.32	7.45	7.3	7.412	7.2	7.24	7.33	7.13	7.1
pO_2_ mmHg (A)	75	68	65	62	116	83.8	74	51.4	89.3
pO_2_ mmHg (X)	70	68	80	35	80	131	71	197	75
pCO_2_ mmHg (A)	21	37	35	33	29	65.9	20	25	15.7
pCO_2_ mmHg (X)	23	37	32	33	25	44.9	22	42	20
HCO_3_ mmol/L (A)	15.2	26	15	17	14	16.1	16	17.9	7.4
HCO_3_ mmol/L (X)	17	26	19	17	14	19.6	16	14.4	10
Lactate mmol/L (A)	4.6	1.1	1.3	1.1	1.2	3.5	1.2	2.5	2
Lactate mmol/L (X)	2.6	1.1	2.6	1.5	1.3	2.2	1.3	8.6	4.5

Normal values: pH (7.34–7.46), pO_2_ (90–113 mmHg), pCO_2_ (34–45 mmHg), HCO_3_ (24–26 mmol/L), and Lactate (less than 2 mmol/L).

**Table 6:** Blood lactate levels at the commencement of TPE.

	Deceased Patients	Survived Patients
Blood lactate level at the beginning of TPE (mmol/L)	8.6; 4.5	2.6; 1.1; 2.6; 1.5; 1.3; 2.2; 1.3

## Data Availability

The datasets used and/or analysed during the current study are available from the corresponding author upon reasonable request.
